# Supplementary material for: A Design Approach to Reducing Stress and Distortion Caused by Adhesive Assembly in Micromachined Deformable Mirrors
Source: Micromachines (Basel). 2023 Mar 27;14(4):740. doi: 10.3390/mi14040740 (PMC10144015; doi:10.3390/mi14040740)
Supplement: Supplementary file 1 [file micromachines-14-00740-s001.zip › micromachines-2301579-supplementary.pdf]

Supporting Information

A Design Approach to Reducing Stress and Distortion Caused by Adhesive Assembly in Micromachined Deformable Mirrors

Wenkuan Man [1,2] and Thomas G. Bifano [1,2,3,*]

[1] Division of Materials Science and Engineering, Boston University, Boston, MA 02215, USA

[2] Mechanical Engineering Department, Boston University, Boston, MA 02215, USA

[3] Photonics Center, Boston University, Boston, MA 02215, USA

* Authors to whom the correspondence should be addressed: tgb@bu.edu

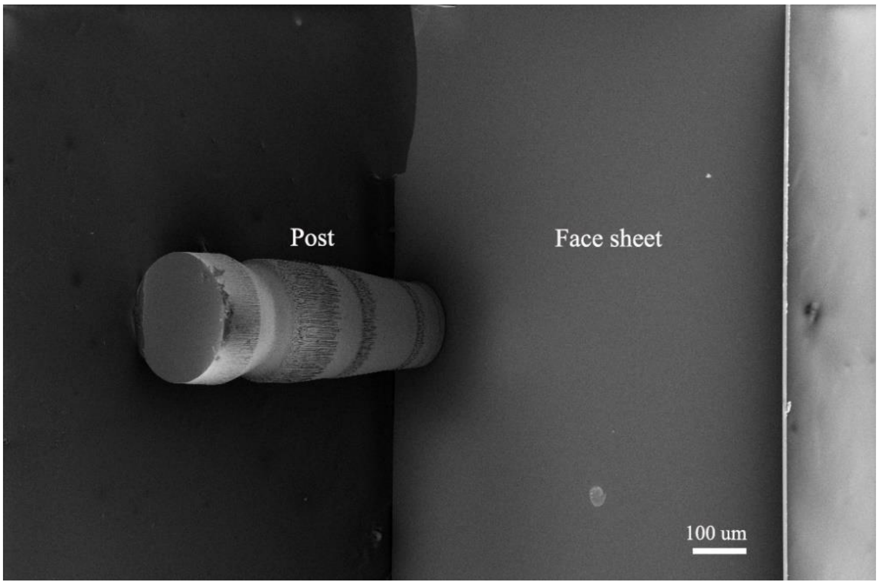

Figure S1. SEM image of single etched silicon post integrated on face sheet.

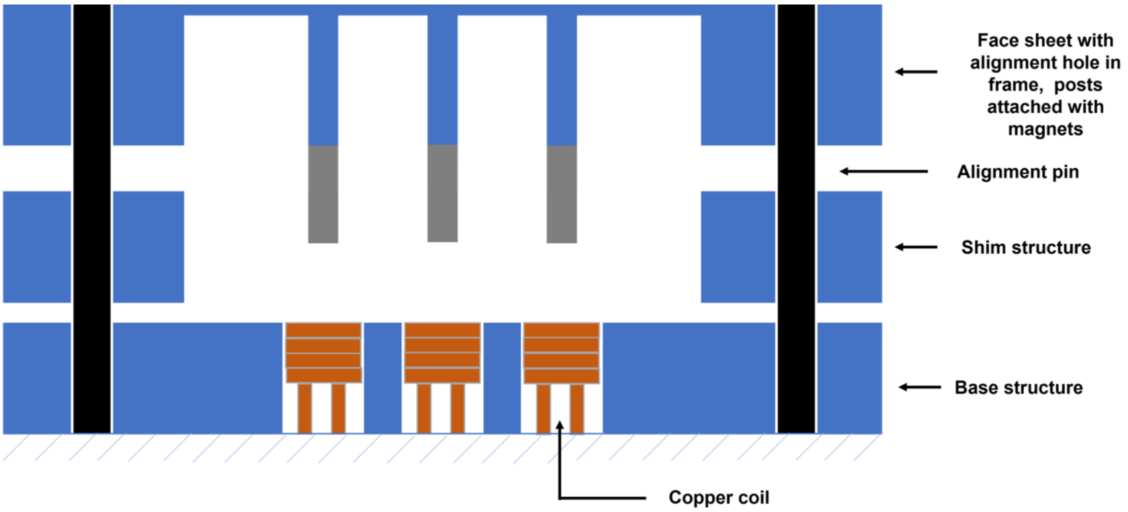

Figure S2. Cross-sectional schematic of the assembly setup for electromagnetic actuation tests. The setup includes three main components layered from top to bottom: A circular face sheet fixed at its edges by a rigid frame, supporting an array of posts (three shown) that have magnets adhesively bonded to their distal ends; a shim material to control the gap between magnets and actuation coils; and a base structure holding the individually addressable copper coils. These three structures are aligned using several pins that connect alignment holes integrated into each layer.

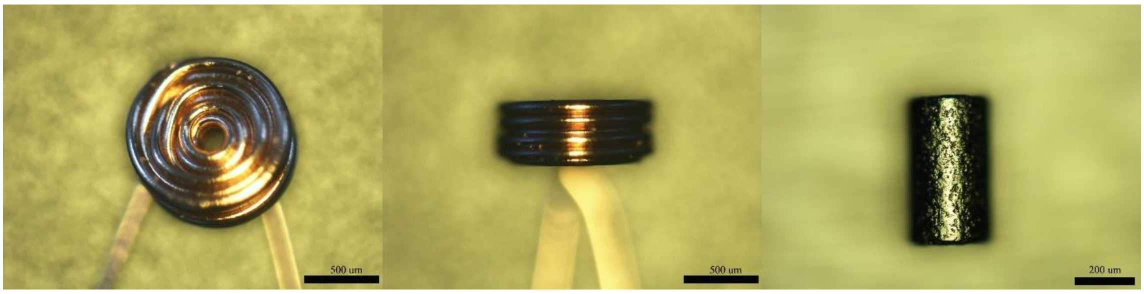

(**a**) (**b**) (**c**)

Figure S3. Microscope photographs of copper coil and magnet used for electromagnetic actuation tests. (**a**) Top view of copper coil; (**b**) Side view of copper coil; (**c**) side view of magnet.
